# Metaphyseal locking compression plate as an external fixator for the distal tibia

**DOI:** 10.1007/s00264-012-1585-7

**Published:** 2012-06-01

**Authors:** Sven A. F. Tulner, Simon D. Strackee, Peter Kloen

**Affiliations:** 1 Department of Orthopaedic Surgery, Academic Medical Center, Meibergdreef 9, 1100 DD Amsterdam, The Netherlands; 2 Department of Plastic and Reconstructive Surgery, Academic Medical Center, Meibergdreef 9, 1100 DD Amsterdam, The Netherlands

## Abstract

**Purpose:**

Recently we coined the term supercutaneous plating using a locking compression plate (LCP) as an external fixator. The use of this technique in peri-articular areas is facilitated by the development of anatomical plates with various screw sizes. The purpose of this report is to describe our results using the metaphyseal locking plate (LCP) as an external fixator in the treatment of infected post-traumatic problems of the distal tibia.

**Methods:**

Between August 2008 and January 2012 a total of seven patients underwent external plating (“supercutaneous plating”) of the distal tibia using a metaphyseal locking plate. Average age was 43 years (range 20–79). Six out of seven patients had a documented infection at the time of external plate application. All patients in this cohort were followed prospectively at regular intervals by the senior author (PK).

**Results:**

The plate was in situ for an average of 17.5 weeks (range 6–60). There were no clinically significant pin site infections. In four patients the plate was kept in place until there was complete consolidation. In three patients the external plate was exchanged for formal internal fixation once the infection had subsided. At the latest follow-up (average 12.8 months, range 4–31), all patients were fully weight bearing with a fully healed tibia. All patients were infection-free with well-healed wounds.

**Conclusion:**

Infection of the distal tibia after treatment of traumatic and post-traumatic problems is a challenging problem. It is common practice that after initial debridement and hardware removal, temporary bony stabilisation is provided by external fixation. Most external frames for the lower leg are bulky and cumbersome, causing significant problems for the patient. To circumvent these issues, we have successfully used an anatomically-contoured metaphyseal locking compression plate as external fixator in a series of seven patients for acute or post-traumatic problems of the tibia.

## Introduction

The soft tissues around the ankle and distal tibia are easily compromised by trauma and subsequent operative fracture treatment [1, 2]. Salvage of these challenging problems more often than not requires a staged treatment based on thorough debridement(s), antibiotic treatment until infection is eliminated followed by reconstruction [3–10]. The initial debridement should include removal of all failed hardware. The resulting instability will compromise the eradication of infection. To prevent this, temporary bony stabilisation can be obtained by some form of external fixation. However, most external frames for the lower leg are bulky and cumbersome for the patient, leading to problems with sleeping, clothing, and can cause an impediment to the contralateral extremity when walking.

Previously, our department reported the use of a dynamic compression plate (DCP) as an external fixator [11, 12]. This involved placement of a nut on the undersurface of the DCP that turned the device into a fixed angle construction. As a modification of this technique, we introduced the standard locking compression plate (LCP, Synthes BV, Zeist, The Netherlands) as an external fixator, and coined the term supercutaneous plating [13]. In this article, we report the successful use of the anatomically-contoured LCP metaphyseal plate as external fixation in seven adult patients for acute or chronic post-traumatic problems in the midshaft and distal tibia.

## Material and methods

Between August 2008 and January 2012 a total of seven patients underwent external plating (“supercutaneous plating”) of the distal tibia using a metaphyseal locking plate. In Table 1 the patient data are summarised. The indications for using external plating were five infected non-unions of the distal tibial and one chronic infection of the tibial shaft. One patient presented late (two weeks after injury) with an open distal tibial fracture and was treated with metaphyseal LCP external fixation as definitive fixation. Average age of the patients was 43 years (range 20–79). Six out of seven patients had a documented infection with known bacteria at the time of external plate application. For one patient there were no culture data but the wound had a purulent discharge. All platings were done by the senior author (PK); the free vascularised fibula was done by the plastic surgeon (SDR).

**Table 1:** Summary of all patients

Patient	Age/sex	Injury	Initial treatment	Infection	Bacteria	External metaphyseal LCP in place (weeks)	Definitive treatment	Outcome	FU (mo)
1	20 M	Pilon fracture	ORIF	Yes	*S. aureus, Enterobacter cloacae*	8	ORIF with LCP, ICBG	Union, infection-free	13
2	35 M	Infected nonunion distal tibia after lengthening	Replating	Yes	*MRSA, P. aeruginosa*	60	Free vascularized fibula transfer	Union, infection-free	14
3	40 M	Grade 2 open pilon fracture	Ex-fix followed by ORIF	Yes	*Enterococcus faecium*	18	ORIF with LCP, ICBG	Union, infection-free	31
4	76 F	Grade 1 open distal tibia fracture	IM nailing	Yes	*S. Aureus*	7	NA	Union, infection-free	9
5	23 M	Grade 2 open distal tibia fracture	Cast	Yes	Unknown	16	NA	Union, infection-free	12
6	56 M	Grade 2 open tibia fracture	Ex-fix followed by ORIF	Yes	*S. aureus, S. marcescens*	8	IM nailing	Union, infection-free	6
7	56 M	Chronic osteomyelitis after osteotomy	NA	Yes	*S. aureus*	6	NA	Infection-free	4

*ORIF* open reduction and internal fixation, *ex-fix* external fixation, *NA* not applicable, *IM* intramedullary, *LCP* locking compression plate, *ICBG* iliac crest bone graft, *FU* follow-up, *M* male, *F* female

All patients in this cohort were followed up prospectively at regular intervals by the senior author (PK). Radiographs were obtained at regular intervals at six weeks, 12 weeks, three months and six months and at final follow-up.

### Surgical technique

With the patient under either general or regional anaesthesia, the involved limb is prepared and draped in the usual standard sterile fashion. No antibiotic treatment is given until deep tissue cultures are obtained. No tourniquet is used; this is to allow intravenous antibiotics to reach the infected area after deep cultures have been taken and to delineate nonvital from vital tissue. The infected area is approached through the old incision. Preoperative consultation with a plastic surgeon should be considered, especially if, at a subsequent stage, a tissue transfer (free vascularised flap or pedicled flap) is planned as part of the reconstructive efforts.

A thorough debridement is performed after removal of all implants that do not provide mechanical stability. Knowledge of the exact type of hardware that is in place is essential to facilitate removal. This should not be taken lightly, as the hardware removal will otherwise be unnecessarily difficult and frustrating.

Soft tissues are generally closed in one layer prior to placement of the LCP as external fixator as the plate might limit easy access for wound closure. Alignment and bony contact is optimised.

Next, a LCP metaphyseal plate for the distal tibia (Synthes BV, Zeist, The Netherlands) of appropriate length is chosen. We recommend relatively long plates to increase the number of options for screw fixation. Also, mechanical stability increases by placing the LCP as close to the bone as possible, yet still allowing for some swelling, and by placement of points of fixation both far and near to the non-union. We only use locking screws that are predrilled with the appropriate size locking drill. Until biomechanical data are available for unicortical vs. bicortical fixation for this plate when used as an external fixator, we prefer bicortical locked screw fixation when we use this plate as an external fixator. For the distal tibia, we use at least three screws (4.5 mm) above and three to four screws (3.5 mm) distally.

Once the wound is closed, the patient can shower after five days with the external fixator in place. Depending on the intrinsic stability of the reconstruction, we let the patient toe-touch or partially weight bear.

## Results

**Fig. 1:**
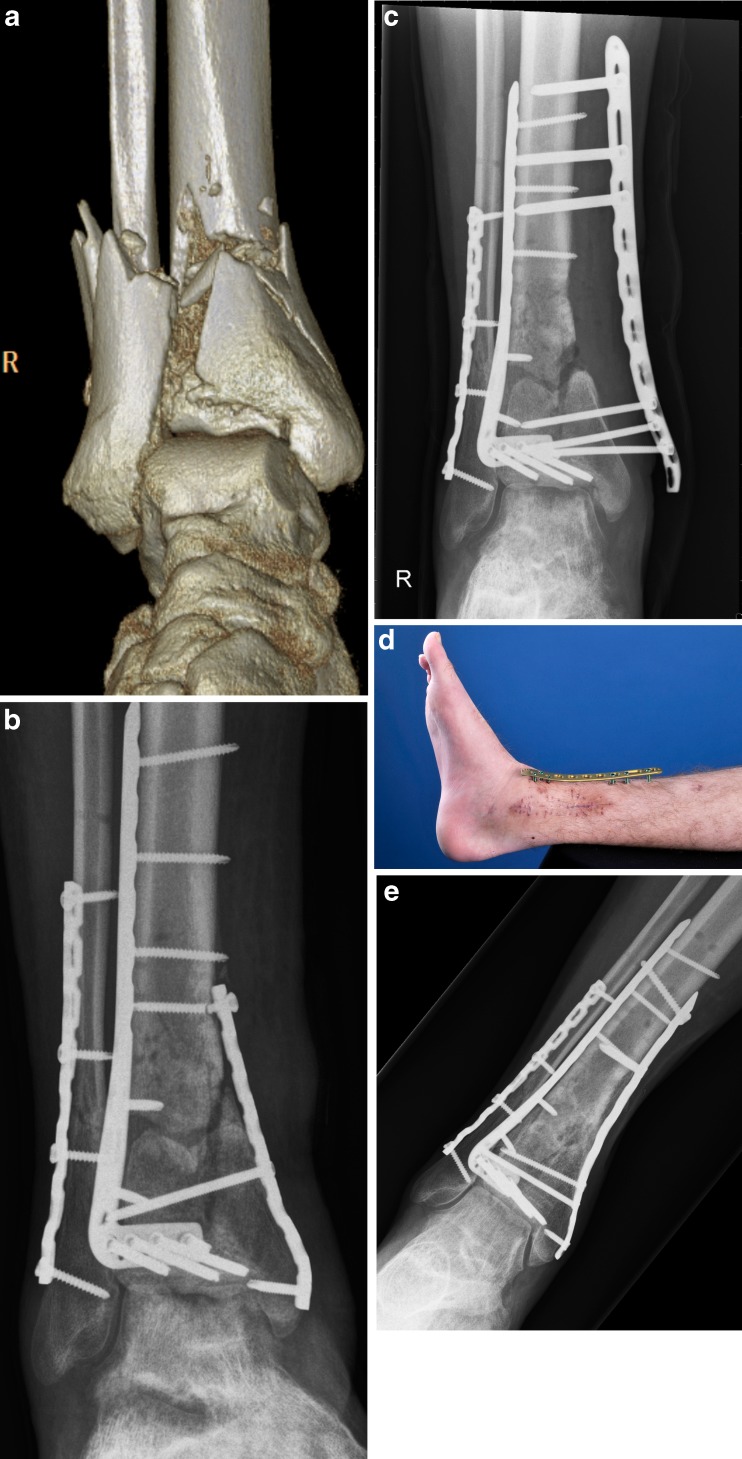
A 19-year-old man presented with a pilon fracture of his right leg (**a**), which was treated by osteosynthesis via an anterolateral approach and a percutaneous medial approach leading to an anatomically reduced joint surface. Four months later, the medial wound showed evidence of infection and delayed union with failure of the medial hardware; notice the broken screw (**b**). The medial plate was removed followed debridement of nonvital bone fragments leaving a large metaphyseal defect stabilised by metaphyseal external LCP fixation (**c**, **d**). After eight weeks of external fixation, the wound healed and his infectious parameters normalised and revision osteosynthesis with bone grafting was performed. Six months later, radiological union was achieved (**e**). His ankle showed a good function and there were no residual signs of infection

**Fig. 2:**
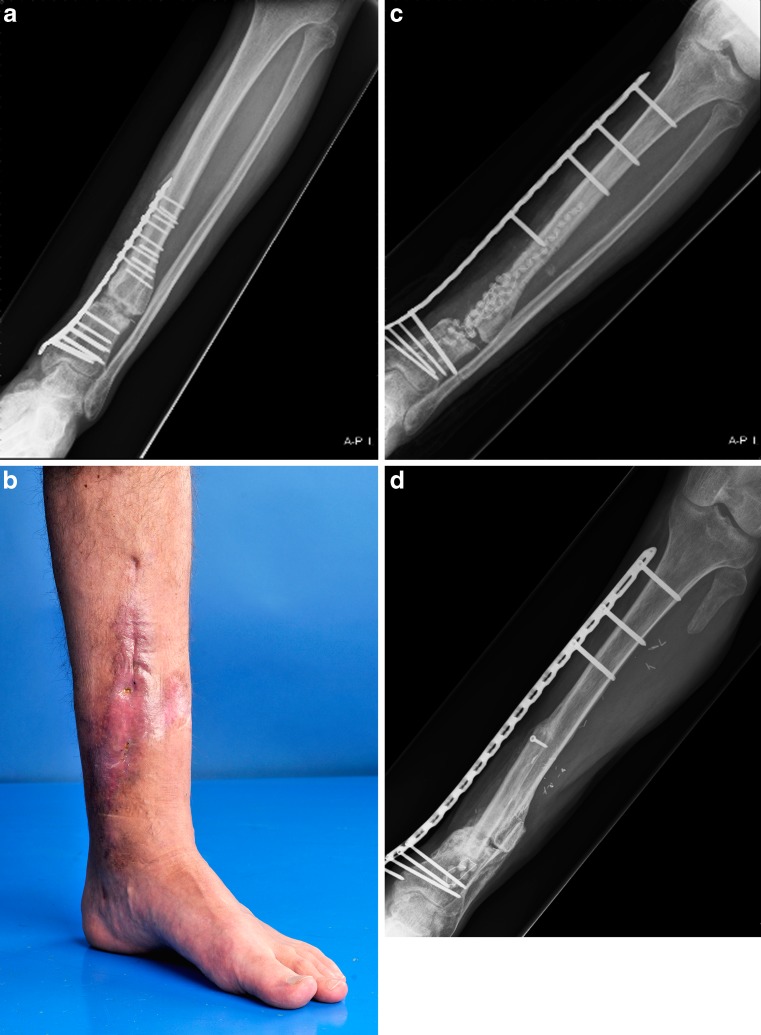
A 41-year-old male presented two years after a distal tibial fracture and failed attempt at a lengthening osteotomy of the tibia. He presented with post-traumatic osteomyelitis (MRSA and *Pseudomonas aeruginosa*) to our hospital for further treatment (**a**, **b**). The patient underwent re-operation with removal of the medial plate followed by thorough debridement and implantation of gentamycin beads followed by stabilisation of the defect by external plate fixation using a metaphyseal LCP (**c**). Five months after placement of the LCP external fixator and three further debridements, a free vascularised fibula transplant was applied in the debrided area by the plastic surgeon. The LCP external plate can easily be concealed under the regular clothing of the patient. Nine months after the free vascularised fibula transfer the tibia had healed (**d**) and there were no residual signs of infection. The plate was removed in the outpatient clinic. The patient was full weightbearing without complaints at the latest follow-up

The plate was in situ for an average of 17.5 weeks (range 6–60). In four patients the plate was left in place until full bony healing was obtained. In three patients the external plate was removed and replaced by formal internal plate (*n* = 2), or reamed intramedullary nail (*n* = 1) fixation. We did not see any significant pin tract infections or any loosening or failure of the hardware. The skin seems to tolerate the titanium screws well and even seems to “adhere” to the screw. At the latest follow-up at an average of 12.8 months (range 4–31), all patients were fully weight bearing with a well-healed tibia. All patients were free of infection without antibiotics with well-healed wounds (See Figs. 1 and 2 for representative cases (case 3 and case 2 respectively from Table 1)).

## Discussion

Standard and circular external fixators for the distal tibia are often bulky and uncomfortable for the patient. Most patients also find them aesthetically unacceptable. External fixation with an anatomically shaped LCP metaphyseal plate as described herein imparts a much lower profile. We were the first to describe the use of LCP as external fixation [13]. More recently others have reported similar experiences [14–17]. The low profile external fixator plate is easily concealed under regular clothing, and there is much less tendency for the frame to strike the contralateral lower leg in the swing-through phase of either leg during ambulation.

From the surgeon’s perspective, the multiple 3.5-mm locking holes distally provide many options for distal fixation, versus the more standard two large external fixator pins. Despite its low profile, external fixation with the metaphyseal LCP seems strong enough to withstand the forces acting on the distal tibia. A possible disadvantage is that its cost tends to be higher than a standard half-pin external fixator (although ring fixators or hybrid fixators are costly as well).

Obviously, our group of patients is relatively small and the indications limited. However, the consistent positive results using this approach support our opinion such that we feel our described use of this plate is easy and very well tolerated by patients. In our hands it has indeed been a useful addition to the techniques used to address these challenging problems.
